# Long‐term effects of a healthy eating blog in mothers and children

**DOI:** 10.1111/mcn.12981

**Published:** 2020-03-05

**Authors:** Audrée‐Anne Dumas, Simone Lemieux, Annie Lapointe, Véronique Provencher, Julie Robitaille, Sophie Desroches

**Affiliations:** ^1^ Centre de recherche Nutrition, Santé et Société (NUTRISS), Institute of Nutrition and Functional Foods, School of Nutrition, Faculty of Agriculture and Food Sciences Université Laval Quebec City Quebec Canada; ^2^ Institute of Nutrition and Functional Foods, Centre de recherche Nutrition, Santé et Société (NUTRISS) Université Laval Quebec City Quebec Canada

**Keywords:** blogs, child, healthy diet, mothers, randomised controlled trial, social media

## Abstract

In the context of low consumption of vegetables and fruits and milk and alternatives among Canadian mothers and children, novel strategies are needed to improve maternal and child nutrition. This study evaluated the long‐term effects of an evidence‐informed healthy eating blog on dietary intakes and food‐related behaviours of mothers and their child. The study presents a secondary outcome analysis of a randomised controlled trial in which 84 mothers (mean age of 37.6 ± 6.7 years) of 2‐ to 12‐year‐old children living in Quebec City, Canada, were randomly assigned to a dietary intervention delivered through a healthy eating blog written by a registered dietitian (RD; *n* = 42) or a control group (*n* = 42) during a period of 6 months. Dietary intakes, maternal eating behaviours, food parenting practices, and body weight were measured at baseline, 3 months, at the end of the intervention (6 months), and 6‐month post‐intervention (12 months). Differences between groups were assessed with mixed linear models. Globally, this study found no evidence of long‐term differences in mean dietary intakes in mothers exposed to the blog and their children as well as other food‐related outcomes and body weight compared with the control condition. Potential predictors of adherence to dietary recommendations in mothers and children (e.g., involvement of children in household food activities) were identified. In conclusion, a healthy eating blog written by an RD did not result in evidence of any long‐term differences in dietary intakes and food‐related behaviours in mothers and their children compared with the control condition.

**Key messages**

- Evidence of efficacy of social media such as blogs to improve dietary behaviours come from short‐term intervention studies, and more research is needed to confirm their efficacy to promote long‐term adherence to dietary recommendations.
- This study showed that a 6‐month blog‐delivered healthy eating intervention written by an RD at a dose of one post per week did not result in long‐term differences in dietary intakes and food‐related behaviours in mothers and children compared with the control condition.
- Potential predictors of sustained healthier diet in mothers and children include self‐efficacy and positive attitude towards family meal planning*,* food conceptualisation skills, and involvement of children in household food activities.

## 1 INTRODUCTION

National dietary recommendations in Canada (Governement of Canada, 2019) advise the consumption of a diet rich in vegetables and fruit, as well as milk and alternatives (e.g., milk, yogurt, and cheese and fortified soy beverages) to achieve a healthy and balanced diet. However, despite sustained public health efforts to promote adherence to dietary recommendations (e.g., Health Canada Eat Well Campaign; Government of Canada, 2015), vegetables and fruit consumption in Canada is lower than recommended (Black & Billette, 2013; Ekwaru et al., 2016). Lower adherence to dietary recommendations is also found for milk and alternatives consumption with especially preoccupying statistics in Canadian children where more than a third of children aged 4 to 9 years old do not consume the recommended daily servings of milk and alternatives each day (Garriguet, 2007). This highlights the need for novel strategies to leverage current public health initiatives to increase vegetables and fruit and milk and alternatives intakes in Canadian adults and children.

Internet users engage with social media every day (Smith & Anderson, 2018), making social media use a norm among various segments of the population. Social media are also used by healthcare professionals such as registered dietitians (RDs; Helm & Jones, 2016) as they represent a unique opportunity to improve knowledge translation in nutrition between RDs, health consumers, and patients. Leveraging this trend, the scientific literature reports a growing number of social media‐delivered interventions promoting healthful lifestyle behaviours such as healthy eating (Dumas, Lapointe, & Desroches, 2018). Evidence regarding the efficacy of social media to improve dietary behaviours mostly come from short‐term intervention studies (Dumas et al., 2018), with limited evidence of maintenance of dietary improvements over follow‐up periods of 3 (Papadaki & Scott, 2008) and 5 months (Choi, Lee, Kang, Lee, & Yoon, 2014). There is thus a need for more research to confirm the efficacy of social media‐delivered interventions to achieve lasting improvements in dietary behaviours.

A variety of individual factors have been suggested to play a role—beyond intervention modalities (Fjeldsoe, Neuhaus, Winkler, & Eakin, 2011)—in the achievement of successful changes in diet and the maintenance of these changes in time (Downer et al., 2016; Hu et al., 2013; Kumanyika et al., 2000). Findings from qualitative studies conducted among mothers also suggest that family settings, preferences of children, and momentary factors (e.g., schedule changes, parental stress, and child mood) could impact food parenting practices (i.e., parenting behaviours related to children's eating, nutrition, or food intakes; Loth, Uy, Neumark‐Sztainer, Fisher, & Berge, 2018; Norman, Nyberg, Elinder, & Berlin, 2018; Spence, Hesketh, Crawford, & Campbell, 2016).

Mothers of young children use social media platforms, including publicly available blogs (defined as web pages in which data entries [posts] are listed in reverse chronological sequence; Herring, Scheidt, Wright, & Sabrina, 2005), to find information about parenting and child feeding (A. E. Doub, Small, & Birch, 2016), seek connections and support from other parents (Pettigrew, Archer, & Harrigan, 2016), and find inspiration for family meals (Allison E. Doub, Small, Levin, LeVangie, & Brick, 2016). Healthy eating blogs thus represent a relevant tool that could be used by health professionals such as RDs to promote lasting improvements among mothers, which could, in turn, be translated into healthier diets in children through improvement in the family food environment. The primary objective of this study was therefore to determine the long‐term effects of a 6‐month dietary intervention delivered through an evidence‐informed healthy eating blog written by an RD on dietary intakes—with a focus on vegetables and fruit and milk and alternatives consumption—diet quality, eating behaviours, food parenting practices, and body weight in Canadian mothers of 2‐ to 12‐year‐old children. The secondary objectives of this study were to examine the long‐term effects of the blog on mothers' children dietary intakes and diet quality and to explore potential predictors of long‐term healthier dietary patterns in mothers and their children. It was hypothesised that mothers exposed to the blog, as well as their children, will show better dietary habits 6 months after the end of the intervention compared with those from a control group with no exposure to the study blog. Findings regarding the short‐term effects of the blog have been reported elsewhere (Dumas et al., 2020).

## 2 METHODS

### 2.1 Study design

This study is a secondary outcome analysis of a parallel, randomised, controlled trial (Dumas et al., 2020) that compared two experimental conditions: (a) a 6‐month intervention delivered through a blog written by an RD who published weekly posts integrating behaviour change techniques and promoting adherence to Canadian dietary recommendations with a focus on vegetables and fruit and milk and alternatives consumption (BLOG group) and (b) a control group with no exposure to the intervention blog. The full protocol of this study is available elsewhere (Dumas et al., 2017). No significant changes were made to the methodology after trial commencement.

### 2.2 Participants and recruitment

Participants of this study were mothers aged 18 years old or over and recruited in Quebec City, Canada, between October 2015 and February 2017, using institutional email lists, flyers, newspapers, Facebook advertisements, and word of mouth. Mothers were eligible if they had an Internet access, had at least one child aged between 2 and 12 years old, were primarily responsible for food purchases and preparation in the household, and consumed fewer than the 2007 edition of Canada's Food Guide's recommended seven servings per day of vegetables and fruit and/or two servings per day of milk and alternatives (Governement of Canada, 2019) as assessed by one dietary recall performed by an RD. Mothers taking medications that could affect their food intake, having an eating disorder, currently dieting, and pregnant or breastfeeding were excluded to eliminate potential confounding variables of dietary habits and body weight change. Eligible mothers met the research coordinator or a graduate student at the research institute located in Quebec City, Canada, to complete self‐administered online questionnaires and in‐person anthropometric measurements for baseline outcome assessment and to sign an informed written consent form. After this baseline visit, enrolled mothers were randomised to one of the experimental groups using a simple randomisation as reported elsewhere (Dumas et al., 2020). Blinding of participants after assignment to intervention groups was not feasible due to the nature of the intervention.

### 2.3 Intervention

Details regarding the development of the intervention blog are reported elsewhere (Dumas et al., 2017). Briefly, mothers randomised to the intervention group (BLOG group) were provided a unique identification code and a password to log on to a private evidence‐informed healthy eating blog, designed in accordance to the preferences of female social media users (Bissonnette‐Maheux et al., 2015; Bissonnette‐Maheux et al., 2017), which aimed to increase intakes of vegetables and fruits and milk and alternatives of mothers and indirectly of their child. During a 6‐month intervention period conducted from January 2016 to September 2017, an RD blogger published one new blog post each week in which positive, nonrestrictive messages integrated theory‐based intervention methods (e.g., modelling, goal setting, and provision of feedback on performance) selected from evidence‐based taxonomies (Kok et al., 2015; S. Michie et al., 2013). Theory‐based intervention methods were selected on the basis of their effectiveness to promote changes in the theoretical constructs predicting the consumption of vegetables and fruit and milk and alternatives in adults (i.e., knowledge, beliefs about consequences [e.g., perceptions of advantages/disadvantages], beliefs about capabilities [e.g., perceptions of barriers/facilitators], and intention and goals; Brewer, Blake, Rankin, & Douglass, 1999; Guillaumie, Godin, & Vezina‐Im, 2010; Kim, Reicks, & Sjoberg, 2003; Park & Ureda, 1999; Wheeler & Chapman‐Novakofski, 2014) and in light of their feasibility of delivery using a blog, as described elsewhere (Dumas et al., 2017). Blog messages also aimed to promote healthy food changes in the home environment and encourage involvement of children in family meal planning and preparation, which, in turn, aimed to improve the quality of children's diet. One to three recipes were published on the blog each week featuring vegetables and fruit and/or milk and alternatives, with explanatory pictures of preparation steps. Additionally, the RD blogger asked open‐ended questions at the end of every blog post to initiate discussions with mothers regarding their realities and perceptions about the advantages/disadvantages and the barriers/facilitators to adopt healthier eating behaviours. Mothers were encouraged to log on to the blog at least once a week and to interact with fellow participants and RD blogger through the comments function of the blog based on individual needs. Comments were posted anonymously on the blog (mothers were named xblog‐X) to encourage participation without apprehensions regarding privacy (Shan et al., 2015). After the intervention, mothers were encouraged to maintain dietary changes in an autonomous way and there was no additional contact with an RD offered. The control group was a control condition with no exposure to the study blog. As for mothers in the BLOG group, mothers in the control group were not offered dietetic counselling during and after the intervention period, but met with the research coordinator to complete all outcome assessments. After the 12‐month follow‐up, those mothers were granted access to the archives of all blog posts and recipes for a period of 1 month. No further benefits were provided to the control group during the intervention period.

### 2.4 Measures

#### 2.4.1 Dietary intakes

The primary outcome of the present paper was the consumption of vegetables and fruit and milk and alternatives of mothers at 12 months, which was reported by “servings” defined on the basis of the 2007 edition of Canada's Food Guide (Governement of Canada, 2019). Vegetables and fruit included all forms of vegetables and fruits, including potatoes and fruit juice, and milk and alternatives included all dairy products (e.g., milk, yogurt, and cheese) and plant‐based milk alternatives (e.g., fortified soy beverages). Dietary and total energy intakes of mothers were measured with three automated, self‐administered web‐based 24‐hr dietary recalls (R24Ws) developed (Jacques et al., 2016) and validated among French–Canadian adults (Lafrenière, Lamarche, Laramée, Robitaille, & Lemieux, 2017) at each assessment time (i.e., baseline, 3 months, after the 6‐month intervention, and 6‐month post‐intervention [12 months]). At each assessment time, R24Ws were randomly assigned to the participants over a period of 1 week to include two weekdays and one weekend day, and data obtained from the three R24Ws were averaged to obtain mean dietary intakes. The database includes 2,865 items linked to the Canadian Nutrition File (version 2015; Health Canada, 2015) or the United States Department of Agriculture, 2015 Nutrient Database for Standard Reference and scored according to different nutritional criteria to enable automated calculation of the Canadian adaptation of the Healthy Eating Index (C‐HEI), which measures diet quality based on conformity with Canada's Food Guide recommendations (Garriguet, 2009). The C‐HEI score was calculated using eight adequacy components (total vegetables and fruit, whole fruit, dark green and orange vegetables, total grain products, whole grains, milk and alternatives, meat and alternatives, and unsaturated fats) and three moderation components (saturated fats, sodium, and “other food”) and ranged from 0 to 100, with higher scores representing better diet quality (Garriguet, 2009). Mothers also completed one 24‐hr recall administered in‐person by an RD with the automated multipass method (Raper, Perloff, Ingwersen, Steinfeldt, & Anand, 2004) for their oldest eligible child (aged 2–12 years old) at each assessment time.

#### 2.4.2 Mothers‐reported meal planning and cooking skills and attitude

Self‐reported skills and attitude of mothers towards meal planning and preparation were measured by a questionnaire adapted by the study authors from the Canadian Community Health Survey Annual Component—Rapid Response on Food Skills, assessing knowledge, planning, and transference of skills (Statistics Canada, 2013a) and on mechanical cooking skills and food conceptualisation (Statistics Canada, 2013b) completed at each assessment time. Self‐reported meal planning skills and enjoyment of planning family meals were measured using 5‐point Likert scales, ranging from 1 (*totally disagree*) to 5 (*totally agree*). Those measures were then grouped to compare the characteristics of mothers who “totally agreed” or “agreed” to those who “totally disagreed,” “disagreed,” or indicated neutral responses. Mothers were asked to classify their meal preparation habits on the basis of the categories described by Statistics Canada based on the types of foods they most often used in the meal of the day that requires the most preparation (i.e., whole/basic ingredients, easy‐to‐prepare items, or take‐up/delivery). Additionally, mothers were questioned on their food conceptualisation skills, namely, if they had ever adjusted a recipe to make it healthier, and, if so, mothers were asked how it was done (i.e., reducing its fat, sugar and/or salt content, adding more vegetables and fruit, or choosing whole grains). Lastly, mothers answered a dichotomous question (yes/no) assessing whether or not their child was involved in grocery shopping, helped with meal preparation, and prepared or cooked meals by himself or herself.

#### 2.4.3 Food parenting practices

Three subscales of the Child Feeding Questionnaire (Birch et al., 2001) were used to measure mothers‐reported use of restriction, pressure to eat, and monitoring of their child's eating at each assessment time.

#### 2.4.4 Eating behaviours

The Three‐Factor Eating Questionnaire (Stunkard & Messick, 1985), a 51‐item validated questionnaire assessing cognitions and behaviours associated with eating (dietary restraint [conscious control of food intake with concerns about shape and weight], disinhibition [overconsumption of food in response to various stimuli associated with a loss of control on food intake], and hunger [food intake in response to feelings and perceptions of hunger]), was completed by mothers at each assessment time. Responses were scored 0 or 1 and summed for each subscale, with higher scores indicating higher levels of dietary restraint, disinhibited eating, and predisposition to hunger, respectively. Intuitive eating was also assessed at each assessment time using the Intuitive Eating Scale (Tylka, 2006), a validated 21‐item questionnaire designed to measure three aspects of intuitive eating: unconditional permission to eat when hungry and what food is desired at the moment, eating for physical rather than emotional reasons, and reliance on internal hunger and satiety cues to determine when and how much to eat. Responses were coded on 5‐point Likert scales for each subscale and averaged to create a total intuitive eating score, with higher scores indicating higher levels of intuitive eating.

#### 2.4.5 Body weight

Body weight was measured to the nearest 0.1 kg in light clothing without shoes (BWB‐800S Digital scale, Tanita) at baseline, 6 months, and 12 months during in‐person clinical appointments at the research institute. Body mass index (kg/m^2^) was calculated according to standardised procedures (Lohman, Roche, & Artorel, 1998).

#### 2.4.6 Engagement with the blog

The total number of logins and postings of comments on the blog use was monitored continuously over the 6‐month intervention using the web analytics service Google Analytics (Google, 2016) and the web analytics plug‐in Slimstat Analytics (Wordpress.org, 2016).

### 2.5 Data analysis

Dietary intakes, food parenting practices, eating behaviours, and body weight measured at different time points were reported using means and standard deviations or standard errors. Differences in responses between groups for measured outcomes were assessed using mixed linear models for repeated measures with group, time, and group by time interaction as fixed effects, and study participants as random effect. In the same model, mean values of measured outcomes in the BLOG group compared with the control group were tested against the null hypothesis by computing the least squares means of fixed effects. When a significant statistical main effect was found, Tukey–Kramer adjusted *p* values were used to identify the precise location of the differences. The skewness in the distribution of all study outcomes was considered, and data were transformed when required to improve the normality of the data distribution; raw data are presented in the tables for transformed variables. Because mixed linear models are robust to data missing at random (Beunckens, Molenberghs, & Kenward, 2005), analyses were conducted without the imputation of missing data and were based on intention‐to‐treat principles. Potential confounders of the outcomes (maternal age; Azagba & Sharaf, 2011), family annual income (Azagba & Sharaf, 2011; Kirkpatrick & Tarasuk, 2007; Ricciuto, Tarasuk, & Yatchew, 2006), ethnicity (Beydoun et al., 2018), education (Azagba & Sharaf, 2011; Ricciuto et al., 2006), body weight (Colapinto, Graham, & St‐Pierre, 2018), total energy intake (Oliveira, Maia, & Lopes, 2014), number of children in their care and children age (Azagba & Sharaf, 2011; Ricciuto et al., 2006) were considered by integrating interaction terms with the main treatment effect into the mixed models. Results presented were based on the most parsimonious models, meaning that only the variables that contributed significantly to variations in any given study outcomes were retained in final models. Cohen's *f* ^*2*^ measure of local effect size for variables within mixed models were calculated according to the procedure described by Selya and colleagues (Selya, Rose, Dierker, Hedeker, & Mermelstein, 2012), with *f* ^*2*^ ≥ 0.02, *f* ^*2*^ ≥ 0.15, and *f* ^*2*^ ≥ 0.35 representing small, moderate, and large effect sizes, respectively. Associations of mothers‐reported attitude and skills towards meal planning and involvement of children in household food activities with the odds of mothers and children meeting dietary recommendations for vegetables and fruit and milk and alternatives at follow‐up were assessed using multiple logistic regression models. The same analyses were performed to explore associations with odds of higher diet quality (defined as the median C‐HEI score of the study sample) in mothers and children. All logistic regression models were adjusted for the previously listed potential cofounders of the outcomes. Sensitivity analyses were performed to test whether an additional adjustment for the experimental condition modified the conclusions of logistic regression models. Based on the results of a previous intervention study (Lanza et al., 2001), a sample size of 82 mothers was estimated to allow the detection of a 28% difference in vegetable intakes at 12 months, with a standard deviation of 2.05 in servings of vegetables, a power of 0.95, and a two‐sided.05 significance level. An attrition rate of 25% was anticipated because attrition rates have been shown to vary between 6% and 75% in social media‐based interventions in dietetic practice (Dumas et al., 2018). Therefore, the planned sample size was 110 mothers; however, this recruitment target was not reached due to longer delays than expected in recruiting mothers meeting inclusion criteria resulting in a final sample of 84 mothers randomised into the study. All analyses were performed using SAS University Edition software (SAS Institute Inc, 2020). For all analyses, the critical *p* value for statistical significance was established at.05 at a 0.95 two‐sided confidence interval (CI).

### 2.6 Ethical considerations

The Université Laval Review Board approved the study protocol (project no. 2014‐257 A‐5/12‐07‐2016). All participants signed an informed written consent form prior to trial onset.

## 3 RESULTS

### 3.1 Study participant characteristics

**Figure 1:**
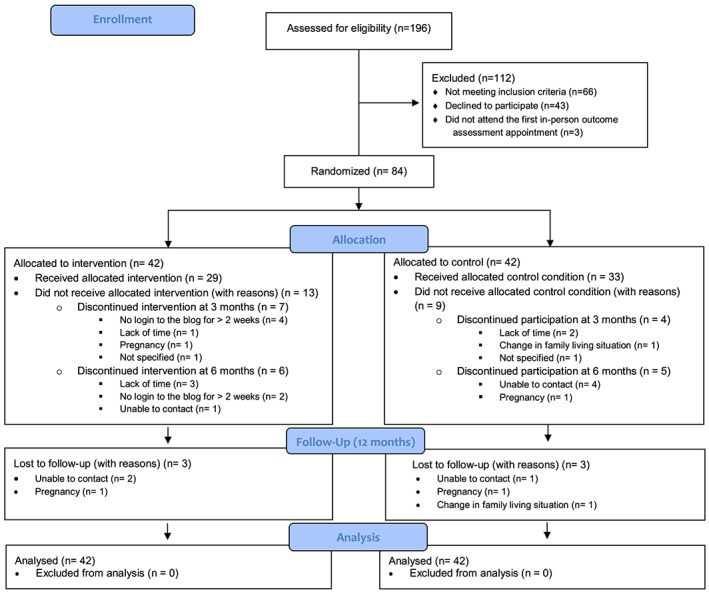
Consolidated Standards of Reporting Trial 2010 (CONSORT) flow diagram of the study [Colour figure can be viewed at wileyonlinelibrary.com]

Figure 1 presents the flow diagram for the study participants. Twenty‐two mothers withdrew from the study prior to the end of the 6‐month dietary intervention (26.2%; BLOG group: *n* = 13; control group: *n* = 9), and six mothers did not complete the 12‐month follow‐up outcome assessment (BLOG group: *n* = 3; control group: *n* = 3) resulting in a retention rate of 66.7% (56/84) at 12 months. The main reasons for withdrawal were inability to contact for outcome assessments, lack of time, new pregnancy, and unforeseen change in family living situations.

Table 1 presents the characteristics of mothers and their child at baseline in terms of age and anthropometric and demographic variables, which were equivalent at baseline between the study groups. Mothers who withdrew from the study (*n* = 28, 33.3%) presented similar demographic characteristics at baseline to those who completed the study until the 12‐month follow‐up (*n* = 56, 66.7%). Exceptions of baseline differences were observed for a higher frequency of mothers with fewer children in their care (*p* < .001), lower annual family income (*p* = .01), and lower C‐HEI scores (mean = 61.5 ± 11.5 and 53.6 ± 14.4 for completers and noncompleters respectively; *p* = .01) among noncompleters.

**Table 1:** Anthropometric and demographic characteristics at baseline of mothers of children aged between 2 and 12 years old randomised to a control (*n* = 42) or an intervention group exposed to an evidence‐informed healthy eating blog for 6 months (*n* = 42)

	BLOG group (*n* = 42)		Control group (*n* = 42)	
Age and anthropometric measures	Mean	*SD*	Mean	*SD*
Age of mothers (years)	37.6	6.9	37.5	6.6
Age of child (years)	7.4	3.2	8.5	3.2
Body weight of mothers (kg)	72.9	13.8	68.1	13.2
Body mass index of mothers (kg/m^2^)	27.1	5.0	25.5	4.7
Sociodemographic characteristics	*n*	%	*n*	%
Number of children in mothers' care				
1	12	29	11	26
2	22	52	18	43
3 or more	8	19	13	31
Child gender				
Girl	22	52	16	38
Boy	20	48	26	62
Ethnicity of mothers^a^				
Caucasian	37	88	39	93
Black	0	0	2	5
Latin	2	5	0	0
Arab	2	5	1	2
Marital status of mothers				
Married or common‐law relationship	34	81	39	93
Separated, divorced, widowed or single	8	19	3	7
Highest level of education completed by mothers				
Secondary	3	7	1	2
College^a^	9	21	11	26
University^a^	30	71	30	71
Working Status of mothers				
Full‐time job or full‐time student	35	83	33	79
Not studying or working full‐time	7	17	9	21
Family income (CAN $)				
0–49,999$	10	24	7	17
50,000–99,999$	15	36	16	38
100,000$ or more	17	41	19	45

Abbreviation: SD, standard deviation.

*n* = 83 due to one missing value.

a In Canada, college refers to a post‐secondary school degree undertaken prior to university.

### 3.2 Effects on dietary intakes and diet quality of mothers and their children

The study did not provide evidence against the null hypothesis of no long‐term difference between the groups in mothers' intakes of vegetables and fruit and milk and alternatives (Table 2); however, the study was able to rule out a difference in mean intakes of vegetables and fruit larger than 1.0 serving (95% CI [−2.1, 1.0]) and a difference in mean intakes of milk and alternatives larger than 0.5 serving (95% CI [−1.0, 0.5]) between groups at 12 months. Effect sizes of the blog for vegetables and fruit and milk and alternatives consumption in mothers across time were small (Cohen's *f* ^2^ = 0.04 for both).

**Table 2:** Intakes of food groups included in Canada's Food Guide and scores of the Canadian adaptation of the Healthy Eating Index in mothers of preschool‐ and school‐aged children randomised in the control group (*n* = 42) and the intervention group exposed to an evidence‐informed healthy eating blog (*n* = 42) at baseline, during (3 months), after the 6‐month dietary intervention (6 months), and 6 months post‐intervention (12 months)

	Control group		BLOG group		Difference BLOG vs. control			Time effect	Group × time interaction
	Mean	*SD*	Mean	*SD*	Adjusted means	*SE*	95% CI	*p* value	*p* value
Vegetables and fruit (servings per day)								.097	.031
Baseline	4.7	1.7	4.9	2.4					
3 months	4.6	2.2	5.7	2.6	0.9	0.5	[−0.5, 2.3]		
6 months	5.1	1.9	6.1^c^	2.4	0.8	0.5	[−0.7, 2.3]		
12 months	5.3	2.1	4.6^c^	2.2	−0.5	0.5	[−2.1, 1.0]		
Vegetables and fruit, excluding fruit juice (servings per day)								.359	.017
Baseline	4.1	1.7	4.2	2.3					
3 months	4.2	2.1	5.1	2.3	0.8	0.5	[−0.6, 2.1]		
6 months	4.3	1.8	5.5^d^	2.3	0.9	0.5	[−0.5, 2.3]		
12 months	4.8	2.1	4.2^d^	2.0	−0.5	0.5	[−2.0, 0.9]		
Vegetables (servings per day)								.129	.013
Baseline	2.7	1.1	2.9	1.7					
3 months	3.0	1.6	3.2	1.4	0.1	0.3	[−0.9, 1.0]		
6 months	3.1	1.4	4.0^e^	1.7	0.7	0.3	[−0.3, 1.7]		
12 months	3.4	1.2	2.9^e^	1.4	−0.5	0.4	[−1.6, 0.5]		
Fruits (servings per day)								.259	.020
Baseline	2.0	1.3	2.0	1.4					
3 months	1.6	1.2	2.5^f^	1.6	0.9^g^	0.3	[0.1, 1.8]		
6 months	2.0	1.2	2.1	1.2	0.2	0.3	[−0.7, 1.1]		
12 months	1.9	1.5	1.8^f^	1.2	0.0	0.3	[−1.0, 0.9]		
Whole fruits (servings per day)								.218	.070
Baseline	1.4	0.9	1.3	1.0					
3 months	1.2	1.0	1.9	1.3	0.8	0.2	[0.1, 1.5]		
6 months	1.2	0.9	1.6	1.0	0.3	0.3	[−0.4, 1.0]		
12 months	1.4	1.3	1.4	1.0	0.0	0.3	[−0.8, 0.8]		
Fruit juice (servings per day)^a^								.419	.568
Baseline	0.6	0.9	0.7	1.0					
3 months	0.4	0.6	0.5	0.8	0.2	0.1	[−0.2, 0.6]		
6 months	0.7	0.9	0.5	0.8	−0.1	0.2	[−0.6, 0.5]		
12 months	0.5	0.9	0.4	0.7	0.1	0.2	[−0.4, 0.5]		
Grain products (servings per day)								.006	.257
Baseline	5.4	2.6	5.5	2.4					
3 months	5.1	2.0	5.1	1.9	0.1	0.4	[−1.0, 1.1]		
6 months	4.9	1.8	4.1	1.9	−0.6	0.4	[−1.8, 0.5]		
12 months	4.9	1.9	5.1	1.8	0.0	0.4	[−1.2, 1.2]		
Whole grain products (servings per day)^a^								.470	.409
Baseline	1.5	1.5	1.6	1.4					
3 months	1.4	1.0	1.6	1.5	0.2	0.3	[−0.6, 1.0]		
6 months	1.6	1.6	1.3	1.3	−0.4	0.3	[−1.3, 0.5]		
12 months	1.4	0.9	1.4	0.9	−0.1	0.3	[−1.1, 0.8]		
Milk and alternatives (servings per day)^b^								.787	.042
Baseline	2.4	1.2	2.2	1.2					
3 months	2.3	1.1	2.3	1.1	0.1	0.2	[−0.5, 0.8]		
6 months	2.0	1.1	2.4	1.1	0.5	0.2	[−0.2, 1.2]		
12 months	2.3	1.1	2.1	1.3	−0.2	0.3	[−1.0, 0.5]		
Meat and alternatives (servings per day)								.156	.142
Baseline	2.3	1.0	2.0	1.0					
3 months	2.1	0.8	2.0	1.0	0.1	0.2	[−0.5, 0.7]		
6 months	2.2	0.9	2.4	0.8	0.3	0.2	[−0.3, 0.8]		
12 months	1.9	0.6	2.3	0.8	0.5	0.1	[0.0, 0.9]		
C‐HEI score								.303	.718
Baseline	61.0	10.6	57.6	14.5					
3 months	62.0	14.6	63.8	13.9	3.6	3.1	[−5.3, 12.5]		
6 months	60.7	11.8	61.4	12.1	0.9	3.3	[−8.6, 10.4]		
12 months	60.3	13.1	61.5	14.3	1.0	3.4	[−8.8, 10.8]		

*Note.* At baseline, *n* = 38 in the control group and *n* = 41 in the BLOG group due to missing data for dietary variables; at 3 months, *n* = 36 in the control group and *n* = 34 in the BLOG group due to missing data for dietary variables; at 6 months, *n* = 31 in the control group and *n* = 29 in the BLOG group due to missing data for dietary variables; and at 12 months, *n* = 28 in the control group and *n* = 27 in the BLOG group due to missing values for dietary variables. *p* values for time and group by time effects were tested against the null hypothesis of no difference between groups in adjusted mixed models. All mixed models were adjusted for baseline value of the outcome variable. In addition, adjustment for potential covariates (total energy intake, maternal age, education, family annual income, ethnicity, body weight, the number of children in their charge, the age of the child, and the body mass index *z* score of the child) was considered only for covariates with *p* < .05 in mixed models.

Abbreviations: C‐HEI, Canadian adaptation of the Healthy Eating Index; CI, confidence interval; SD, standard deviation; SE, standard error.

a Log‐transformed data.

b Foods included in the milk and alternatives category include milk (evaporated, powered, skim, 1%, 2%, whole, chocolate, goat), fortified soy beverages, yogurt and yogurt drinks, cheese, buttermilk, kefir, paneer, and pudding/custard (made with milk).

c Within‐group difference between 6 months and the 12‐month follow‐up with *p* < .05 (adjusted mean difference at 12 months, −1.2 serving per day; 95% CI [−0.1, −2.3]).

d Within‐group difference between 6 months and the 12‐month follow‐up with *p* < .05 (adjusted mean difference at 12 months, −1.1 serving per day; 95% CI [−0.04, −2.2]).

e Within‐group difference between 6 months and the 12‐month follow‐up with *p* < .05 (adjusted mean difference at 12 months, −1.0 serving per day; 95% CI [−0.1, −1.9]).

f Within‐group difference between 3 months and the 12‐month follow‐up with *p* < .05 (adjusted mean difference at 12 months, −0.8 serving per day; 95% CI [−0.03, −1.5]).

g Between‐group difference with *p* < .05.

Within the BLOG group, mean intakes of vegetables and fruit and mean intakes of vegetables decreased by 1.2 serving per day (95% CI [−0.1, −2.3]; *p* = .02) and by 1.0 serving per day (95% CI [−0.1, −1.9]; *p* = .01), respectively, between the end of the intervention (6 months) and the 12‐month follow‐up. At 3 months, there was a higher mean intake of fruits of 0.9 serving per day in the BLOG group compared with the control group (95% CI [0.1, 1.8]; *p* = .03). Within the BLOG group, fruit consumption decreased by 0.8 serving per day between 3 months and the 12‐month follow‐up (95% CI [−0.03, −1.5]; *p* = .04). The study did not provide evidence of any long‐term difference between the groups in mothers' mean intakes of total grain products, whole grain products, meat and alternatives intakes, and C‐HEI scores in mothers with a 0.95 two‐sided confidence interval (Table 2).

For children, the study did not provide evidence against the null hypothesis of no long‐term difference between the groups for mean intakes of vegetables and fruit and milk and alternatives, as well as for intakes of other food groups of Canada's Food Guide and C‐HEI scores with a 0.95 two‐sided confidence interval (Table 3).

**Table 3:** Intakes of food groups included in Canada's Food Guide and scores of the Canadian adaptation of the Healthy Eating Index in preschool‐ and school‐aged children of mothers randomised in the control group (*n* = 42) and the intervention group exposed to an evidence‐informed healthy eating blog (*n* = 42) at baseline, during (3 months), after the 6‐month dietary intervention (6 months), and 6 months post‐intervention (12 months)

	Control group		BLOG group		Difference BLOG vs. control			Time effect	Group × time interaction
	Mean	*SD*	Mean	*SD*	Adjusted means	*SE*	95% CI	*p* value	*p* value
Vegetables and fruit (servings per day)								.293	.601
Baseline	5.0	2.0	5.1	2.3					
3 months	5.7	2.8	5.8	2.6	0.0	0.6	[−1.7, 1.7]		
6 months	5.8	2.6	6.0	3.0	0.1	0.6	[−1.6, 1.9]		
12 months	5.7	2.8	5.1	1.7	−0.6	0.6	[−2.5, 1.3]		
Vegetables and fruit, excluding fruit juice (servings per day)								.351	.643
Baseline	4.3	2.2	4.2	2.1					
3 months	4.5	2.1	4.9	2.4	0.3	0.5	[−1.1, 1.8]		
6 months	4.9	2.2	5.2	2.7	0.2	0.5	[−1.3, 1.7]		
12 months	4.7	2.2	4.6	1.7	−0.3	0.5	[−1.9, 1.3]		
Vegetables (servings per day)^a^								.215	.836
Baseline	2.7	1.8	2.4	1.9					
3 months	2.6	1.4	2.6	1.6	0.1	0.4	[−1.0, 1.2]		
6 months	3.0	1.8	3.3	2.0	0.4	0.4	[−0.7, 1.5]		
12 months	2.7	1.4	2.7	1.1	0.0	0.4	[−1.3, 1.2]		
Fruits (servings per day)^a^								.178	.979
Baseline	2.3	1.1	2.7	1.5					
3 months	3.1	2.2	3.1	2.0	−0.2	0.4	[−1.5, 1.0]		
6 months	2.9	2.2	2.7	1.5	−0.3	0.5	[−1.7, 1.0]		
12 months	3.0	2.3	2.5	1.2	−0.6	0.5	[−2.0, 0.8]		
Whole fruits (servings per day)^a^								.634	.452
Baseline	1.6	1.0	1.9	1.2					
3 months	2.0	1.5	2.3	1.6	0.1	0.3	[−0.8, 1.1]		
6 months	2.0	1.5	1.9	1.3	−0.3	0.3	[−1.2, 0.7]		
12 months	2.0	1.4	1.9	1.2	−0.2	0.4	[−1.3, 0.8]		
Fruit juice (servings per day)^a^								.142	.337
Baseline	0.7	0.8	0.8	1.0					
3 months	1.1	1.5	0.8	1.2	−0.4	0.3	[−1.1, 0.4]		
6 months	0.9	1.4	0.8	1.0	0.0	0.3	[−0.8, 0.8]		
12 months	1.0	1.3	0.5	0.9	−0.3	0.3	[−1.2, 0.6]		
Grain products (servings per day)								.904	.259
Baseline	5.3	2.6	4.8	2.0					
3 months	5.2	2.1	4.6	2.3	−0.7	0.5	[−2.2, 0.8]		
6 months	4.9	2.0	4.6	2.3	−0.4	0.5	[−1.9, 1.2]		
12 months	4.8	2.0	5.3	2.2	0.5	0.6	[−1.2, 2.2]		
Whole grain products (servings per day)^a^								.939	.904
Baseline	1.3	1.5	1.1	1.5					
3 months	1.5	1.5	0.9	1.3	−0.5	0.3	[−1.5, 0.4]		
6 months	1.3	1.4	1.1	1.2	−0.3	0.3	[−1.3, 0.6]		
12 months	1.4	1.3	1.4	1.3	−0.2	0.4	[−1.3, 0.9]		
Milk and alternatives (servings per day)^a^^,^ b								.175	.093
Baseline	2.5	1.2	2.5	1.5					
3 months	2.8	1.6	2.6	1.4	−0.3	0.3	[−1.2, 0.5]		
6 months	2.6	1.3	3.0	1.8	0.4	0.3	[−0.5, 1.3]		
12 months	2.7	1.6	2.1	1.4	−0.6	0.3	[−1.6, 0.4]		
Meat and alternatives (servings per day)								.070	.800
Baseline	1.5	1.0	1.5	1.0					
3 months	1.4	0.9	1.5	0.8	0.0	0.2	[−0.6, 0.7]		
6 months	1.6	0.8	1.9	1.1	0.2	0.2	[−0.5, 0.9]		
12 months	1.6	1.2	1.8	1.2	0.0	0.3	[−0.7, 0.8]		
C‐HEI score^b^								.134	.544
Baseline	67.7	13.5	64.6	13.7					
3 months	69.4	11.3	67.5	13.8	−1.6	3.0	[−10.1, 7.0]		
6 months	68.3	10.8	71.4	13.3	3.0	3.1	[−6.0, 12.0]		
12 months	65.5	15.6	66.3	14.5	−0.2	3.3	[−9.8, 9.3]		

*Note.* At baseline, *n* = 42 and *n* = 42 in the control group and in the BLOG group, respectively; at 3 months, *n* = 34 in the control group and *n* = 33 in the BLOG group due to missing data for dietary variables; at 6 months, *n* = 31 in the control group and *n* = 30 in the BLOG group due to missing data for dietary variables; and at 12 months, *n* = 29 in the control group and *n* = 25 in the BLOG group due to missing data for response variables. *p* values for time and group by time effects were tested against the null hypothesis in adjusted mixed models. All mixed models were adjusted for baseline value of the outcome variable and experimental phases. In addition, adjustment for potential covariates (total energy intake, maternal age, education, family annual income, ethnicity, body weight, the number of children in mothers' care, the age of the child, and the body mass index *z* score of the child) was considered only when covariates were shown to be significant at *p* < .05 in mixed models.

Abbreviations: C‐HEI, Canadian adaptation of the Healthy Eating Index; CI, confidence interval; SD, standard deviation; SE, standard error.

a Log‐transformed data.

b Foods included in the milk and alternatives category include milk (evaporated, powered, skim, 1%, 2%, whole, chocolate, goat), fortified soy beverages, yogurt and yogurt drinks, cheese, buttermilk, kefir, paneer, and pudding/custard (made with milk).

### 3.3 Effects on body weight, food parenting practices, and eating behaviours of mothers

The study did not provide evidence of long‐term difference between the groups for mothers' mean body weight (adjusted mean difference at 12 months, 0.4 kg; 95% CI [−2.6, 3.3]; group by time interaction effect, *p* = .75), mothers' use of monitoring (adjusted mean difference at 12 months, 0.3; 95% CI [−0.4, 1.0]; group by time interaction effect, *p* = .19), restriction (adjusted mean difference at 12 months, −0.1; 95% CI [−0.7, 0.4]; group by time interaction effect, *p* = .91), and pressure to eat (adjusted mean difference at 12 months, −0.3; 95% CI [−0.9, 0.3]; group by time interaction effect, *p* = .37), as well as for mothers' levels of dietary restraint (adjusted mean difference at 12 months, 1.0; 95% CI [−0.8, 2.8]; group by time interaction effect, *p* = .32), disinhibition (adjusted mean difference at 12 months, −0.1; 95% CI [−1.5, 1.3]; group by time interaction effect, *p* = .97), susceptibility to hunger (adjusted mean difference at 12 months, −0.3; 95% CI [−2.1, 1.6]; group by time interaction effect, *p* = .88), and total intuitive eating scores (adjusted mean difference at 12 months, 0.0; 95% CI [−0.3, 0.3]; group by time interaction effect, *p* = .37).

### 3.4 Predictors of healthier dietary patterns in mothers and children at follow‐up

Merging both intervention groups, at the 12‐month follow‐up, mothers who reported feeling competent and enjoying planning family meals had higher odds of consuming at least five servings per day of vegetables and fruit and at least two servings per day of milk and alternatives (Table 4). Planning enough vegetables and fruits for preparing meals, cooking from mostly basic ingredients, and food conceptualisation skills to improve the nutritional content of recipes—including the addition of vegetables and fruits—were globally not associated with healthier dietary patterns in mothers at 12 months. Exceptions were found for mothers who reported having reduced the fat content of a recipe in the past, as they were more likely to consume at least two servings per day of milk and alternatives and for mothers who reported having reduced the sugar content of a recipe in the past, as they were more likely to have higher C‐HEI scores at 12 months compared with mothers who reported having never made those adjustments to recipes. Within the BLOG group, a frequency of logins on the blog higher than or equal to the median number of logins of the group (≥25 logins) over the course of the intervention predicted higher odds of higher C‐HEI scores at 12 months. In contrast, the publication of comments on the blog was not associated with any of the dietary variables in mothers.

**Table 4:** Associations between self‐perceived meal planning skills and attitude, meal preparation habits, food conceptualisation skills, engagement with the blog and the consumption of vegetables and fruit and milk and alternatives, and scores of the Canadian adaptation of the Healthy Eating Index (C‐HEI) at follow‐up (12 months) in the 84 mothers of children aged between 2 and 12 years old randomised in the study

Mothers dietary intakes at 12 months												
Vegetables and fruit consumption, ≥5 servings per day					Milk and alternatives consumption, ≥2 servings per day				Diet quality, C‐HEI ≥ 61.2			
Unadjusted			Adjusted^a^		Unadjusted		Adjusted^a^		Unadjusted		Adjusted^a^	
OR		95% CI	OR	95% CI	OR	95% CI	OR	95% CI	OR	95% CI	OR	95% CI
Meal planning skills and attitude												
Feels competent planning family meal												
No	1.0		1.0		1.0		1.0		1.0		1.0	
Yes	3.7	[1.0, 14.1]	4.3	[0.9, 21.5]	4.8*	[1.3, 17.4]	18.8^b^, **	[2.6, 137.4]	0.5	[0.1, 1.7]	1.1	[0.2, 5.5]
Enjoys planning family meals												
No	1.0		1.0		1.0		1.0		1.0		1.0	
Yes	2.8	[0.8, 9.8]	5.8*	[1.2, 29.6]	1.8	[0.5, 5.8]	5.9*	[1.1, 32.1]	0.6	[0.2, 2.1]	1.1	[0.2, 4.7]
Plans enough vegetables and fruits for meal preparation												
No	1.0		1.0		1.0		1.0		1.0		1.0	
Yes	2.6	[0.5, 14.4]	2.7	[0.4, 17.9]	2.2	[0.5, 10.3]	4.4	[0.6, 32.4]	0.4	[0.1, 2.4]	0.2	[0.0, 2.0]
Meal preparation habits												
Cooks from mostly whole/basic ingredients												
No	1.0		1.0		1.0		1.0		1.0		1.0	
Yes	2.0	[0.5, 7.8]	1.8	[0.4, 9.3]	2.3	[0.6, 8.5]	4.0	[0.7, 22.8]	3.3	[0.9, 12.6]	4.1	[0.7, 25.0]
Food conceptualisation skills												
Has adjusted a recipe to make it healthier by reducing its salt content												
No	1.0		1.0		1.0		1.0		1.0		1.0	
Yes	0.7	[0.2, 2.2]	0.6	[0. 2, 2.2]	0.9	[0.3, 2.8]	0.8	[0.2, 3.2]	1.0	[0.4, 2.8]	0.9	[0.3, 3.0]
Has adjusted a recipe to make it healthier by reducing its fat content												
No	1.0		1.0		1.0		1.0		1.0		1.0	
Yes	1.5	[0.5, 4.2]	1.6	[0.5, 5.3]	4.2*	[1.4, 13.0]	6.3*	[1.5, 25.9]	1.6	[0.6, 4.1]	1.7	[0.6, 5.0]
Has adjusted a recipe to make it healthier by reducing its sugar content												
No	1.0		1.0		1.0		1.0		1.0		1.0	
Yes	1.1	[0.3, 3.6]	1.2	[0.3, 5.0]	3.2	[0.9, 10.9]	3.1	[0.7, 14.4]	3.3*	[1.3, 8.3]	3.7*	[1.2, 11.1]
Has adjusted a recipe to make it healthier by adding whole grains												
No	1.0		1.0		1.0		1.0		1.0		1.0	
Yes	1.2	[0.4, 3.5]	1.1	[0.3, 3.5]	1.1	[0.4, 3.1]	1.1	[0.3, 3.8]	0.9	[0.4, 2.4]	1.3	[0.4, 3.8]
Has adjusted a recipe to make it healthier by adding more vegetables and fruits												
No	1.0		1.0		1.0		1.0		1.0		1.0	
Yes	1.1	[0.3, 3.3]	1.0	[0.2, 4.0]	1.1	[0.3, 3.3]	0.6	[0.1, 2.7]	1.4	[0.6, 3.5]	1.5	[0.5, 4.3]
Engagement with the blog^c^												
Frequency of logins on the blog												
< 25.5	1.0		1.0		1.0		1.0		1.0		1.0	
≥ 25.5	0.9	[0.2, 4.4]	0.1	[0.0, 3.3]	0.5	[0.1, 2.2]	0.0	[<0.001, 1.3]	2.1	[0.5, 8.8]	8.5*	[1.0, 74.0]
Frequency of posted comments on the blog												
< 3.5	1.0		1.0		1.0		1.0		1.0		1.0	
≥ 3.5	1.4	[0.3, 6.6]	0.1	[0.0, 7.2]	1.2	[0.3, 5.6]	1.4	[0.1, 20.9]	2.1	[0.5, 8.8]	4.9	[0.6, 40.0]
Posted at least one comment on the blog												
No	1.0		1.0		1.0		1.0		1.0		1.0	
Yes	0.3	[0.0, 1.7]	0.1	[0.0, 4.3]	0.9	[0.1, 5.6]	2.4	[0.1, 40.4]	3.5	[0.6, 21.2]	5.2	[0.4, 63.2]

Abbreviations: CI, confidence interval; OR, odds ratio.

a Results for multivariate logistic (*n* = 46 due to missing data for the response or explanatory variables) adjusted for age of the mother, family living status, education, ethnicity, annual family income, working status, the number of children in their care, and the age of the child.

b Use estimate with caution because of high variability in the coefficient of variation.

BLOG group only, *n* = 27 for univariate analyses and *n* = 26 for multivariate analyses due to missing data for the response or explanatory variables.

* *p* < .05.

** *p* < .01.

For children, participating in grocery shopping and helping prepare meals were associated with higher odds of higher C‐HEI scores at 12 months (Table 5). In mothers and children, similar conclusions were observed for all associations when an additional statistical adjustment for the experimental group (BLOG vs control group) was performed (data not shown).

**Table 5:** Associations between involvement of children in household food activities and consumption of vegetables and fruit and milk and alternatives and scores of the Canadian adaptation of the Healthy Eating Index (C‐HEI) at follow‐up (12 months) in 2‐ to 12‐year‐old children of the 84 mothers randomised in the study

	Children dietary intakes at 12 months											
	Vegetables and fruit consumption, ≥5 servings per day				Milk and alternatives consumption, ≥2 servings per day				Diet quality, HEI ≥ 66.75			
	Unadjusted		Adjusted^a^		Unadjusted		Adjusted^a^		Unadjusted		Adjusted^a^	
	OR	95% CI	OR	95% CI	OR	95% CI	OR	95% CI	OR	95% CI	OR	95% CI
Children involvement in household food activities												
Make suggestions to family meals												
No	1.0		1.0		1.0		1.0		1.0		1.0	
Yes	2.5	[0.4, 15.4]	4.0	[0.3, 58.8]	0.9	[0.1, 5.5]	1.0	[0.1, 8.0]	2.8	[0.5, 17.3]	3.2	[0.3, 31.2]
Participate in grocery shopping												
No	1.0		1.0		1.0		1.0		1.0		1.0	
Yes	2.0	[0.6, 6.5]	1.5	[0.4, 6.2]	1.4	[0.4, 4.6]	2.8	[0.6, 13.1]	3.5*	[1.0, 11.8]	1.7	[0.4, 8.6]
Help prepare meals												
No	1.0		1.0		1.0		1.0		1.0		1.0	
Yes	1.1	[0.3, 3.7]	0.8	[0.2, 3.0]	2.1	[0.6, 7.3]	2.1	[0.5, 8.7]	2.9	[0.9, 9.8]	7.3*	[1.2, 45.8]
Prepare meals or cook foods by themselves												
No	1.0		1.0		1.0		1.0		1.0		1.0	
Yes	0.9	[0.3, 3.0]	1.2	[0.3, 5.3]	1.7	[0.5, 6.1]	1.6	[0.4, 7.3]	0.7	[0.2, 2.4]	1.9	[0.4, 9.6]

Abbreviations: CI, confidence interval; OR, odds ratio.

a Results for multivariate logistic (*n* = 45 due to missing data for the response or explanatory variables) adjusted for age of the mother, family living status, education, ethnicity, annual family income, working status, the number of children in their care, and the age of the child.

* *p* < .05.

## 4 DISCUSSION

This study presented the long‐term effects of a 1‐year randomised, parallel, controlled trial that evaluated the effects of a 6‐month exposure to a healthy eating blog written by an RD, used as a stand‐alone strategy in a real‐word setting, on dietary intakes, eating behaviours, food parenting practices, and body weight of mothers. This study also reported secondary outcomes regarding the long‐term effects of the blog on the dietary intakes of mothers' preschool‐ and school‐aged children. Globally, there was no evidence of long‐term differences in mean dietary intakes and food‐related behaviours between mothers exposed to the blog and their children compared with the control condition, which contrasts with the a priori hypothesis, but is consistent with the short‐term intervention findings (Dumas et al., 2020).

The absence of evidence of long‐term differences in dietary outcomes between mothers exposed to the blog and those in the control condition is consistent with previous research showing that, without additional support, acute intervention effects tend to diminish or treatment differences vanish over time (Beresford et al., 2010; MacKinnon et al., 2010; Toobert, Strycker, Barrera, & Glasgow, 2010). Mothers were encouraged to maintain their post‐intervention dietary changes in an autonomous matter as they were not offered any dietetic counselling during the 6‐month period that followed the end of the intervention. Interventions achieving successful maintenance of behaviour change including dietary changes (defined as statistically significant differences between groups at follow‐up) have been shown to share specific characteristics including face‐to‐face contacts and use of many behaviour change strategies (i.e., more than six) during the intervention and employing follow‐up prompts (Fjeldsoe et al., 2011). In the digital health behaviour change research field, the equation appears to be complexified by methodological challenges that researchers and clinicians have to tackle such as low levels of adherence and high levels of attrition (Eysenbach, 2005; Susan Michie, Yardley, West, Patrick, & Greaves, 2017; Corneel Vandelanotte et al., 2016). Currently, few trials have provided support for the long‐term effectiveness of social media‐delivered interventions on dietary behaviour change (Maher et al., 2014; Williams, Hamm, Shulhan, Vandermeer, & Hartling, 2014).

One explanation for the absence of evidence of any long‐term differences in dietary outcomes between the groups may be that the dose of the intervention (i.e., one blog post per week) was insufficient to produce lasting dietary changes, given the fact that mothers engage with various other social media platforms every day such as Facebook, Instagram, and Pinterest (Duggan, Lenhart, & Ellison, 2015). Exploratory analysis showed that more frequent logins on the blog were associated with higher C‐HEI scores in mothers exposed to the healthy eating blog. This suggests that a more extensive use of the blog (in contrast to simply being granted access to the blog and using it sparingly) could reflect a greater motivation to change dietary behaviours and curiosity about the content of the blog (Brouwer et al., 2009) and, in turn, may increase the efficacy of blog‐delivered healthy eating interventions. In contrast, posting comments on the blog did not predict healthier dietary patterns in mothers. Abstention from posting comments on healthy eating blogs is common (Bissonnette‐Maheux et al., 2015; Caplette et al., 2017; Partridge et al., 2015), and it is not necessarily representative of lower motivation levels to improve dietary behaviours. Indeed, a myriad of factors influences the motivations to actively participate in online communities by posting comments, such as group identity and individual personality traits (Sun, Rau, & Ma, 2014). It was beyond the scope of this study to explore these factors.

Vandelanotte and colleagues (C. Vandelanotte et al., 2018; C. Vandelanotte & Maher, 2015) have argued that ecological study designs are essential to examine how digital health interventions work in real life or ecologically valid settings outside of controlled research environments. Nowadays, an increasing number of RDs write healthy eating blogs available to the public to disseminate evidence‐based nutrition knowledge and showcase healthy foods and recipes on social media (Dietitians of Canada, 2018; Helm & Fromm, 2018; Mortensen & Ferguson, 2016). It is possible that mothers who follow RD bloggers for a sustained period of time (e.g., for one or several years), even if it is not on a regular basis, could become more empowered to try new foods and make healthy changes in their family's diet. Future studies should examine the mechanisms of the effects of exposure to healthy eating blogs on long‐term healthy dietary patterns such as potential collection of small changes in psychological constructs predicting behaviour change such as nutritional knowledge, attitude, self‐efficacy, and motivation.

Lastly, findings from logistic regression analysis gave some insights regarding potentially effective strategies to include in future interventions aiming to improve maternal and child diet. Self‐efficacy and positive attitude towards family meal planning as well as some food conceptualisation skills, such as reducing the fat and sugar content of recipes to make them healthier, were associated with higher likelihood of healthier dietary patterns in mothers at follow‐up including the consumption of at least five servings per day of vegetables and fruit and the consumption of at least two servings per day of milk and alternatives. Involvement of children in household food activities, such as participating in grocery shopping and helping prepare meals, were associated with higher likelihood of better diet quality in children at follow‐up. This is consistent with previous cross‐sectional and intervention studies showing positive associations between children's involvement in those activities and healthy eating such as higher consumption of vegetables (van der Horst, Ferrage, & Rytz, 2014), fruits (Lavelle et al., 2016), diet quality (Chu, Storey, & Veugelers, 2014), and willingness to try unfamiliar foods containing vegetables (Allirot, Maiz, & Urdaneta, 2018). Given that children primarily learn to cook with their mothers (Lavelle et al., 2016), future social media‐delivered interventions that are specifically designed to improve hands‐on food and cooking skills of mothers should be investigated as potential public health strategies to promote sustained healthy eating among families.

### 4.1 Limits of the research

This study was subject to some limitations that must be acknowledged. First, the small sample size, consequent to longer delays than expected in recruiting eligible mothers, may have limited the ability to draw conclusions regarding the acute and long‐term effectiveness of the blog to improve mothers' and children's diet. Second, blog engagement was measured with usage metrics alone (i.e., total number of logins and postings of comments) and thus cannot capture the multidimensional aspect of this construct (Perski, Blandford, West, & Michie, 2017; Short et al., 2018). A qualitative assessment of mothers' perceptions and experience with the blog could have helped increase the understanding of facilitators and barriers regarding their use of the blog and complement study findings regarding the associations between blog use and dietary behaviour changes. Lastly, the study sample was mostly composed of highly educated and socioeconomically advantaged Caucasian mothers. Given the growing popularity of social media use among a wide range of demographic groups (Smith & Anderson, 2018), further research is needed to determine if healthy eating blogs could be an effective knowledge translation strategy to improve the diet of socioeconomically disadvantaged and ethnically heterogeneous populations.

## 5 CONCLUSIONS

In conclusion, this study found no evidence of any long‐term differences in dietary intakes, body weight, and food‐related behaviours in mothers exposed to a 6‐month blog‐delivered healthy eating intervention written by an RD at a dose of one post per week and their children compared with a control condition as assessed 6 months following the end of the intervention. However, some individual factors predicting better adherence to dietary recommendations and higher diet quality in mothers and children, such as self‐efficacy and positive attitude towards family meal planning as well as involvement of children in meal preparation, were identified. Other studies will be needed to examine the impact of using healthy eating blogs during longer periods of time and at a higher dose of exposure, in ecologically valid settings where they coexist alongside various nutrition influences available online and in the food environment, and to investigate the mechanisms of their effects on the maintenance of healthy dietary changes.

## CONFLICTS OF INTEREST

The authors declare that they have no competing interests.

## CONTRIBUTIONS

AAD contributed to the acquisition of data and was in charge of the analysis and interpretation of data and drafting of the manuscript. AL coordinated the acquisition of data. JR, SL, and VP contributed to the conception and design of the study as well as to the interpretation of data, and SD was in charge of the conception and design of the study and had primary responsibility for the final content. All of the authors critically reviewed the manuscript and approved its final version.

## References

[mcn12981-bib-0001] Allirot, X. , Maiz, E. , & Urdaneta, E. (2018). Shopping for food with children: A strategy for directing their choices toward novel foods containing vegetables. Appetite, 120(Supplement C), 287–296. 10.1016/j.appet.2017.09.008 28918160

[mcn12981-bib-0002] Azagba, S. , & Sharaf, M. F. (2011). Disparities in the frequency of fruit and vegetable consumption by socio‐demographic and lifestyle characteristics in Canada. Nutrition Journal, 10, 118 10.1186/1475-2891-10-118 22027238 PMC3217867

[mcn12981-bib-0003] Beresford, S. A. , Thompson, B. , Bishop, S. , Macintyre, J. , McLerran, D. , & Yasui, Y. (2010). Long‐term fruit and vegetable change in worksites: Seattle 5 a Day follow‐up. American Journal of Health Behavior, 34(6), 707–720. 10.5993/ajhb.34.6.7 20604696 PMC3658284

[mcn12981-bib-0004] Beunckens, C. , Molenberghs, G. , & Kenward, M. G. (2005). Direct likelihood analysis versus simple forms of imputation for missing data in randomized clinical trials. Clinical Trials, 2(5), 379–386. 10.1191/1740774505cn119oa 16315646

[mcn12981-bib-0005] Beydoun, M. A. , Fanelli‐Kuczmarski, M. T. , Beydoun, H. A. , Dore, G. A. , Canas, J. A. , Evans, M. K. , & Zonderman, A. B. (2018). Dairy product consumption and its association with metabolic disturbance in a prospective study of urban adults. The British Journal of Nutrition, 119(6), 706–719. 10.1017/s0007114518000028 29553032 PMC5863589

[mcn12981-bib-0006] Birch, L. L. , Fisher, J. O. , Grimm‐Thomas, K. , Markey, C. N. , Sawyer, R. , & Johnson, S. L. (2001). Confirmatory factor analysis of the Child Feeding Questionnaire: A measure of parental attitudes, beliefs and practices about child feeding and obesity proneness. Appetite, 36(3), 201–210. 10.1006/appe.2001.0398 11358344

[mcn12981-bib-0007] Bissonnette‐Maheux, V. , Dumas, A. A. , Provencher, V. , Lapointe, A. , Dugrenier, M. , Straus, S. , … S. (2017). Women's perceptions of usefulness and ease of use of four healthy eating blog characteristics: A qualitative study of 33 French–Canadian women. Journal of the Academy of Nutrition and Dietetics, 118, 1220 10.1016/j.jand.2017.08.012 1227.e3 29107587

[mcn12981-bib-0008] Bissonnette‐Maheux, V. , Provencher, V. , Lapointe, A. , Dugrenier, M. , Dumas, A. A. , Pluye, P. , … S. (2015). Exploring women's beliefs and perceptions about healthy eating blogs: A qualitative study. Journal of Medical Internet Research, 17(4), e87 10.2196/jmir.3504 25858777 PMC4407018

[mcn12981-bib-0009] Black, J. L. , & Billette, J. M. (2013). Do Canadians meet Canada's Food Guide's recommendations for fruits and vegetables? Applied Physiology, Nutrition, and Metabolism, 38(3), 234–242. 10.1139/apnm-2012-0166 23537013

[mcn12981-bib-0010] Brewer, J. L. , Blake, A. J. , Rankin, S. A. , & Douglass, L. W. (1999). Theory of reasoned action predicts milk consumption in women. Journal of the American Dietetic Association, 99(1), 39–44. 10.1016/S0002-8223(99)00012-7 9917730

[mcn12981-bib-0011] Brouwer, W. , Oenema, A. , Crutzen, R. , de Nooijer, J. , de Vries, N. K. , & Brug, J. (2009). What makes people decide to visit and use an internet‐delivered behavior‐change intervention?: A qualitative study among adults. Health Education, 109(6), 460–473. 10.1108/09654280911001149

[mcn12981-bib-0012] Caplette, M. E. , Provencher, V. , Bissonnette‐Maheux, V. , Dugrenier, M. , Lapointe, A. , Gagnon, M. P. , … S. (2017). Increasing fruit and vegetable consumption through a healthy eating blog: A feasibility study. JMIR research protocols, 6(4), e59 10.2196/resprot.6622 28420600 PMC5413798

[mcn12981-bib-0013] Choi, Y. , Lee, M. J. , Kang, H. C. , Lee, M. S. , & Yoon, S. (2014). Development and application of a web‐based nutritional management program to improve dietary behaviors for the prevention of metabolic syndrome. Computers, Informatics, Nursing, 32(5), 232–241. 10.1097/CIN.0000000000000054 24651253

[mcn12981-bib-0014] Chu, Y. L. , Storey, K. E. , & Veugelers, P. J. (2014). Involvement in meal preparation at home is associated with better diet quality among Canadian children. Journal of Nutrition Education and Behavior, 46(4), 304–308. 10.1016/j.jneb.2013.10.003 24238908

[mcn12981-bib-0015] Colapinto, C. K. , Graham, J. , & St‐Pierre, S. (2018). Trends and correlates of frequency of fruit and vegetable consumption, 2007 to 2014. Health Reports, 29(1), 9–14. 29341026

[mcn12981-bib-0016] Dietitians of Canada . (2018). Member blogs. Retrieved from https://www.dietitians.ca/Media/Member-Blogs.aspx

[mcn12981-bib-0017] Doub, A. E. , Small, M. , & Birch, L. (2016). An exploratory analysis of child feeding beliefs and behaviors included in food blogs written by mothers of preschool‐aged children. Journal of Nutrition Education and Behavior, 48(2), 93–103 e101. 10.1016/j.jneb.2015.09.001 26601887

[mcn12981-bib-0018] Doub, A. E. , Small, M. L. , Levin, A. , LeVangie, K. , & Brick, T. R. (2016). Identifying users of traditional and Internet‐based resources for meal ideas: An association rule learning approach. Appetite, 103, 128–136. 10.1016/j.appet.2016.04.006 27067739

[mcn12981-bib-0019] Downer, M. K. , Gea, A. , Stampfer, M. , Sanchez‐Tainta, A. , Corella, D. , Salas‐Salvado, J. , … Martinez‐Gonzalez, M. A. (2016). Predictors of short‐ and long‐term adherence with a Mediterranean‐type diet intervention: The PREDIMED randomized trial. International Journal of Behavioral Nutrition and Physical Activity, 13, 67 10.1186/s12966-016-0394-6 27297426 PMC4907003

[mcn12981-bib-0020] Duggan, M. , Lenhart, A. , Lampe, C. , & Ellison, N. B. (2015). Parents and social media. Retrieved from http://www.pewinternet.org/2015/07/16/parents-and-social-media/

[mcn12981-bib-0021] Dumas, A. A. , Lapointe, A. , & Desroches, S. (2018). Users, uses, and effects of social media in dietetic practice: Scoping review of the quantitative and qualitative evidence. Journal of Medical Internet Research, 20(2), e55 10.2196/jmir.9230 29463487 PMC5840482

[mcn12981-bib-0022] Dumas, A. A. , Lemieux, S. , Lapointe, A. , Provencher, V. , Robitaille, J. , & Desroches, S. (2017). Development of an evidence‐informed blog to promote healthy eating among mothers: Use of the intervention mapping protocol. JMIR research protocols, 6(5), e92 10.2196/resprot.7147 28526669 PMC5457529

[mcn12981-bib-0023] Dumas, A. A. , Lemieux, S. , Lapointe, A. , Provencher, V. , Robitaille, J. , & Desroches, S. (2020). Effects of an evidence‐informed healthy eating blog on dietary intakes and food‐related behaviors of mothers of preschool‐ and school‐aged children: A randomized controlled trial. Journal of the Academy of Nutrition and Dietetics, 120(1), 53–68. 10.1016/j.jand.2019.05.016 31519466

[mcn12981-bib-0024] Ekwaru, J. P. , Ohinmaa, A. , Loehr, S. , Setayeshgar, S. , Thanh, N. X. , & Veugelers, P. J. (2016). The economic burden of inadequate consumption of vegetables and fruit in Canada. Public Health Nutrition, 20(3), 515–523. 10.1017/S1368980016002846 27819197 PMC5426323

[mcn12981-bib-0025] Eysenbach, G. (2005). The law of attrition. Journal of Medical Internet Research, 7(1), e11 10.2196/jmir.7.1.e11 15829473 PMC1550631

[mcn12981-bib-0026] Fjeldsoe, B. , Neuhaus, M. , Winkler, E. , & Eakin, E. (2011). Systematic review of maintenance of behavior change following physical activity and dietary interventions. Health Psychology, 30(1), 99–109. 10.1037/a0021974 21299298

[mcn12981-bib-0027] Garriguet, D. (2007). Canadians' eating habits. Health Reports, 18(2), 17–32. 17578013

[mcn12981-bib-0028] Garriguet, D. (2009). Diet quality in Canada. Health Reports, 20(3), 41–52. 19813438

[mcn12981-bib-0029] Google . (2016). Google analytics. Retrieved from https://www.google.ca/analytics/

[mcn12981-bib-0030] Governement of Canada . (2019). Canada's food guide. Retrieved from https://food-guide.canada.ca/en/

[mcn12981-bib-0031] Government of Canada . (2015). Healthy eating. Retrieved from http://healthycanadians.gc.ca/eating-nutrition/healthy-eating-saine-alimentation/index-eng.php

[mcn12981-bib-0032] Guillaumie, L. , Godin, G. , & Vezina‐Im, L. A. (2010). Psychosocial determinants of fruit and vegetable intake in adult population: A systematic review. International Journal of Behavioral Nutrition and Physical Activity, 7, 12 10.1186/1479-5868-7-12 20181070 PMC2831029

[mcn12981-bib-0033] Health Canada . Canadian Nutrient File (CNF), 2015 Retrieved from https://food-nutrition.canada.ca/cnf-fce/index-eng.jsp

[mcn12981-bib-0034] Helm, J. , & Fromm, L. (2018). Nutrition blog network: Powered by dietitians. Retrieved from http://www.nutritionblognetwork.com/

[mcn12981-bib-0035] Helm, J. , & Jones, R. M. (2016). Practice paper of the academy of nutrition and dietetics: Social media and the dietetics practitioner: Opportunities, challenges, and best practices. Journal of the Academy of Nutrition and Dietetics, 116(11), 1825–1835. 10.1016/j.jand.2016.09.003 27788767

[mcn12981-bib-0036] Herring, S. , Scheidt, L. A. , Wright, E. , & Sabrina, B. (2005). Weblogs as a bridging genre. Information Technology & People, 18(2), 142–171.

[mcn12981-bib-0037] Hu, E. A. , Toledo, E. , Diez‐Espino, J. , Estruch, R. , Corella, D. , Salas‐Salvado, J. , … M. A. (2013). Lifestyles and risk factors associated with adherence to the Mediterranean diet: A baseline assessment of the PREDIMED trial. PLoS ONE, 8(4), e60166 10.1371/journal.pone.0060166 23637743 PMC3639284

[mcn12981-bib-0038] Jacques, S. , Lemieux, S. , Lamarche, B. , Laramée, C. , Corneau, L. , & Lapointe, A. (2016). Development of a web‐based 24‐h dietary recall for a French–Canadian population. Nutrients, 8 10.3390/nu8110724 PMC5133109 27854276

[mcn12981-bib-0039] Kim, K. , Reicks, M. , & Sjoberg, S. (2003). Applying the theory of planned behavior to predict dairy product consumption by older adults. Journal of Nutrition Education and Behavior, 35(6), 294–301. 10.1016/s1499-4046(06)60343-6 14642214

[mcn12981-bib-0040] Kirkpatrick, S. , & Tarasuk, V. (2007). The relationship between low income and household food expenditure patterns in Canada. Public Health Nutrition, 6(6), 589–597. 10.1079/PHN2003517 14690040

[mcn12981-bib-0041] Kok, G. , Gottlieb, N. H. , Peters, G. Y. , Mullen, P. D. , Parcel, G. S. , Ruiter, R. A. , … Bartholomew, L. K. (2015). A taxonomy of behaviour change methods: An intervention mapping approach. Health Psychology Review, 1–16. 10.1080/17437199.2015.1077155 26262912 PMC4975080

[mcn12981-bib-0042] Kumanyika, S. K. , Bowen, D. , Rolls, B. J. , Van Horn, L. , Perri, M. G. , Czajkowski, S. M. , & Schron, E. (2000). Maintenance of dietary behavior change. Health Psychology, 19(1, Suppl), 42–56. 10.1037/0278-6133.19.Suppl1.42 10709947

[mcn12981-bib-0043] Lafrenière, J. , Lamarche, B. , Laramée, C. , Robitaille, J. , & Lemieux, S. (2017). Validation of a newly automated web‐based 24‐hour dietary recall using fully controlled feeding studies. BMC Nutrition, 3(1), 34 10.1186/s40795-017-0153-3 10 32153814 PMC7050885

[mcn12981-bib-0044] Lanza, E. , Schatzkin, A. , Daston, C. , Corle, D. , Freedman, L. , Ballard‐Barbash, R. , … PPT Study Group (2001). Implementation of a 4‐y, high‐fiber, high‐fruit‐and‐vegetable, low‐fat dietary intervention: Results of dietary changes in the Polyp Prevention Trial. The American Journal of Clinical Nutrition, 74(3), 387–401. 10.1093/ajcn/74.3.387 11522565

[mcn12981-bib-0045] Lavelle, F. , Spence, M. , Hollywood, L. , McGowan, L. , Surgenor, D. , McCloat, A. , … M. (2016). Learning cooking skills at different ages: A cross‐sectional study. International Journal of Behavioral Nutrition and Physical Activity, 13(1), 119 10.1186/s12966-016-0446-y 27842556 PMC5109777

[mcn12981-bib-0046] Lohman, T. , Roche, A. , & Artorel, R. (1998). The Airlie (VA) Consensus conference: Standardization of anthropometric measurements. Champaign, IL, USA: Human Kinetics Publishers.

[mcn12981-bib-0047] Loth, K. A. , Uy, M. , Neumark‐Sztainer, D. , Fisher, J. O. , & Berge, J. M. (2018). A qualitative exploration into momentary impacts on food parenting practices among parents of pre‐school aged children. Appetite, 130, 35–44. 10.1016/j.appet.2018.07.027 30059769 PMC6472478

[mcn12981-bib-0048] MacKinnon, D. P. , Elliot, D. L. , Thoemmes, F. , Kuehl, K. S. , Moe, E. L. , Goldberg, L. , … Ranby, K. W. (2010). Long‐term effects of a worksite health promotion program for firefighters. American Journal of Health Behavior, 34(6), 695–706. 10.5993/ajhb.34.6.6 20604695

[mcn12981-bib-0049] Maher, C. A. , Lewis, L. K. , Ferrar, K. , Marshall, S. , De Bourdeaudhuij, I. , & Vandelanotte, C. (2014). Are health behavior change interventions that use online social networks effective? A systematic review. Journal of Medical Internet Research, 16(2), e40 10.2196/jmir.2952 24550083 PMC3936265

[mcn12981-bib-0050] Michie, S. , Richardson, M. , Johnston, M. , Abraham, C. , Francis, J. , Hardeman, W. , … C. E. (2013). The behavior change technique taxonomy (v1) of 93 hierarchically clustered techniques: Building an international consensus for the reporting of behavior change interventions. Annals of Behavioral Medicine, 46(1), 81–95. 10.1007/s12160-013-9486-6 23512568

[mcn12981-bib-0051] Michie, S. , Yardley, L. , West, R. , Patrick, K. , & Greaves, F. (2017). Developing and evaluating digital interventions to promote behavior change in health and health care: Recommendations resulting from an international workshop. Journal of Medical Internet Research, 19(6), e232 10.2196/jmir.7126 28663162 PMC5509948

[mcn12981-bib-0052] Mortensen, A. , & Ferguson, M. (2016). The guide to dietitians' social media habits. Retrieved from http://appetitecommunications.com.au/wp-content/uploads/2016/12/ACDC-2016-Guide-to-Dietitians-Social-Media-Habits.pdf.

[mcn12981-bib-0053] Norman, A. , Nyberg, G. , Elinder, L. S. , & Berlin, A. (2018). Parental strategies for influencing the diet of their children—A qualitative study from disadvantaged areas. Appetite, 125, 502–511. 10.1016/j.appet.2018.03.002 Parental strategies for influencing the diet of their children ‐ A qualitative study from disadvantaged areas 29524473

[mcn12981-bib-0054] Oliveira, A. , Maia, B. , & Lopes, C. (2014). Determinants of inadequate fruit and vegetable consumption amongst Portuguese adults. Journal of human nutrition and dietetics, 27(s2), 194–203. 10.1111/jhn.12143 23889074

[mcn12981-bib-0055] Papadaki, A. , & Scott, J. (2008). Follow‐up of a web‐based tailored intervention promoting the Mediterranean diet in Scotland. Patient education and counseling, 73(2), 256–263. 10.1016/j.pec.2008.05.030 18640000

[mcn12981-bib-0056] Park, K. , & Ureda, J. R. (1999). Specific motivations of milk consumption among pregnant women enrolled in or eligible for WIC. Journal of Nutrition Education Behavior, 31(2), 76–85. 10.1016/S0022-3182(99)70399-7

[mcn12981-bib-0057] Partridge, S. R. , McGeechan, K. , Hebden, L. , Balestracci, K. , Wong, A. T. , Denney‐Wilson, E. , … M. (2015). Effectiveness of a mHealth lifestyle program with telephone support (TXT2BFiT) to prevent unhealthy weight gain in young adults: Randomized controlled trial. JMIR mHealth and uHealth, 3(2), e66 10.2196/mhealth.4530 26076688 PMC4526939

[mcn12981-bib-0058] Perski, O. , Blandford, A. , West, R. , & Michie, S. (2017). Conceptualising engagement with digital behaviour change interventions: A systematic review using principles from critical interpretive synthesis. Translational Behavioral Medicine, 7(2), 254–267. 10.1007/s13142-016-0453-1 27966189 PMC5526809

[mcn12981-bib-0059] Pettigrew, S. , Archer, C. , & Harrigan, P. (2016). A thematic analysis of mothers' motivations for blogging. Maternal and Child Health Journal, 20(5), 1025–1031. 10.1007/s10995-015-1887-7 26662282

[mcn12981-bib-0060] Raper, N. , Perloff, B. , Ingwersen, L. , Steinfeldt, L. , & Anand, J. (2004). An overview of USDA's Dietary Intake Data System. Journal of Food Composition and Analysis, 17, 545–555. 10.1016/j.jfca.2004.02.013

[mcn12981-bib-0061] Ricciuto, L. , Tarasuk, V. , & Yatchew, A. (2006). Socio‐demographic influences on food purchasing among Canadian households. European Journal of Clinical Nutrition, 60(6), 778–790. 10.1038/sj.ejcn.1602382 16418741

[mcn12981-bib-0062] SAS Institute Inc . (2020). SAS University Edition, version 3.71. Cary, NC, USA.

[mcn12981-bib-0063] Selya, A. S. , Rose, J. S. , Dierker, L. C. , Hedeker, D. , & Mermelstein, R. J. (2012). A practical guide to calculating Cohen's f(2), a measure of local effect size, from PROC MIXED. Frontiers in Psychology, 3, 111 10.3389/fpsyg.2012.00111 22529829 PMC3328081

[mcn12981-bib-0064] Shan, L. C. , Panagiotopoulos, P. , Regan, Á. , De Brún, A. , Barnett, J. , Wall, P. , & McConnon, Á. (2015). Interactive communication with the public: Qualitative exploration of the use of social media by food and health organizations. Journal of Nutrition Education and Behavior, 47(1), 104–108. 10.1016/j.jneb.2014.09.004 25449827

[mcn12981-bib-0065] Short, C. E. , DeSmet, A. , Woods, C. , Williams, S. L. , Maher, C. , Middelweerd, A. , … Crutzen, R. (2018). Measuring engagement in eHealth and mHealth behavior change interventions: Viewpoint of methodologies. Journal of Medical Internet Research, 20(11), e292 10.2196/jmir.9397 30446482 PMC6269627

[mcn12981-bib-0066] Smith, A. , & Anderson, M. (2018). Social media use in 2018. Retrieved from http://www.pewinternet.org/2018/03/01/social-media-use-in-2018/

[mcn12981-bib-0067] Spence, A. C. , Hesketh, K. D. , Crawford, D. A. , & Campbell, K. J. (2016). Mothers' perceptions of the influences on their child feeding practices—A qualitative study. Appetite, 105, 596–603. 10.1016/j.appet.2016.06.031 27352882

[mcn12981-bib-0068] Statistics Canada . (2013a). *Canadian Community Health Survey (CCHS) Rapid Response on Food Skills (part 1) knowledge, planning and transference of skills complement to the user guide* . Retrieved from http://www23.statcan.gc.ca/imdb-bmdi/instrument/3226_Q4_V1-eng.pdf:

[mcn12981-bib-0069] Statistics Canada . (2013b). *Canadian Community Health Survey (CCHS) Rapid Response on Food Skills (part 2) mechanical skills and food conceptualisation complement to the user guide* . Retrieved from http://www23.statcan.gc.ca/imdb-bmdi/instrument/3226_Q4_V2-eng.pdf:

[mcn12981-bib-0070] Stunkard, A. J. , & Messick, S. (1985). The three‐factor eating questionnaire to measure dietary restraint, disinhibition and hunger. Journal of Psychosomatic Research, 29(1), 71–83. 3981480 10.1016/0022-3999(85)90010-8

[mcn12981-bib-0071] Sun, N. , Rau, P. P.‐L. , & Ma, L. (2014). Understanding lurkers in online communities: A literature review. Computers in Human Behavior, 38, 110–117. 10.1016/j.chb.2014.05.022

[mcn12981-bib-0072] Toobert, D. J. , Strycker, L. A. , Barrera, M. , & Glasgow, R. E. (2010). Seven‐year follow‐up of a multiple‐health‐behavior diabetes intervention. American Journal of Health Behavior, 34(6), 680–694. 20604694 10.5993/ajhb.34.6.5 PMC3065935

[mcn12981-bib-0073] Tylka, T. L. (2006). Development and psychometric evaluation of a measure of intuitive eating. Journal of Counseling Psychology, 53(2), 226–240. 10.1037/0022-0167.53.2.226

[mcn12981-bib-0074] United States Department of Agriculture . (2015). USDA National Nutrient Database for Standard 4nce. Retrieved from https://ndb.nal.usda.gov/ndb/search

[mcn12981-bib-0075] van der Horst, K. , Ferrage, A. , & Rytz, A. (2014). Involving children in meal preparation. Effects on food intake. Appetite, 79, 18–24. 10.1016/j.appet.2014.03.030 24709485

[mcn12981-bib-0076] Vandelanotte, C. , Duncan, M. J. , Kolt, G. S. , Caperchione, C. M. , Savage, T. N. , Van Itallie, A. , … Mummery, W. K. (2018). More real‐world trials are needed to establish if web‐based physical activity interventions are effective. British Journal of Sports Medicine. 10.1136/bjsports-2018-099437 29970409

[mcn12981-bib-0077] Vandelanotte, C. , & Maher, C. A. (2015). Why we need more than just randomized controlled trials to establish the effectiveness of online social networks for health behavior change. American Journal of Health Promotion, 30(2), 74–76. 10.4278/ajhp.141204-CIT-605 26517586

[mcn12981-bib-0078] Vandelanotte, C. , Müller, A. M. , Short, C. E. , Hingle, M. , Nathan, N. , Williams, S. L. , … Maher, C. A. (2016). Past, present, and future of eHealth and mHealth research to improve physical activity and dietary behaviors. Journal of nutrition education and behavior, 48(3), 219–228.e211. 10.1016/j.jneb.2015.12.006 26965100

[mcn12981-bib-0079] Wheeler, A. , & Chapman‐Novakofski, K. (2014). Women infant and children program participants' beliefs and consumption of soy milk: Application of the theory of planned behavior. Nutrition research and practice, 8(1), 66–73. 10.4162/nrp.2014.8.1.66 24611108 PMC3944159

[mcn12981-bib-0080] Williams, G. , Hamm, M. P. , Shulhan, J. , Vandermeer, B. , & Hartling, L. (2014). Social media interventions for diet and exercise behaviours: A systematic review and meta‐analysis of randomised controlled trials. BMJ Open, 4(2), e003926 10.1136/bmjopen-2013-003926 PMC3927930 24525388

[mcn12981-bib-0081] Wordpress.org (2016). Plugin directory: WP Slimstat Analytics. Retrieved from https://wordpress.org/plugins/wp-slimstat/

